# Interventional Treatments of Colorectal Liver Metastases Using Thermal Ablation and Transarterial Chemoembolization: A Single-Center Experience over 26 Years

**DOI:** 10.3390/cancers16091756

**Published:** 2024-04-30

**Authors:** Thomas J. Vogl, Jason Freichel, Tatjana Gruber-Rouh, Nour-Eldin Abdelrehim Nour-Eldin, Wolf-Otto Bechstein, Stefan Zeuzem, Nagy N. N. Naguib, Ulrich Stefenelli, Hamzah Adwan

**Affiliations:** 1 Clinic for Radiology and Nuclear Medicine, University Hospital Frankfurt, Goethe University, 60590 Frankfurt am Main, Germany; 2 Department of Diagnostic and Interventional Radiology, Faculty of Medicine, Cairo University, Cairo 12613, Egypt; 3 Department of General and Visceral Surgery, University Hospital Frankfurt, Goethe-University, 60590 Frankfurt am Main, Germany; 4 Department of Internal Medicine I, University Hospital Frankfurt, Goethe University-Frankfurt am Main, 60590 Frankfurt am Main, Germany; 5 Radiology Department, AMEOS Hospital Halberstadt, 38820 Halberstadt, Germany; 6 Department of Diagnostic and Interventional Radiology, Faculty of Medicine, Alexandria University, Alexandria 21526, Egypt; 7 Statistical Analysis Dr. Stefenelli, Untere Bockgasse 5, 97070 Würzburg, Germany

**Keywords:** colorectal cancer liver metastasis, interventional oncological treatment, thermal ablation, laser-induced thermotherapy, microwave ablation, transarterial chemoembolization

## Abstract

**Simple Summary:**

Colorectal cancer is one of the most commonly diagnosed cancers worldwide with a high probability of developing metastasis over the course of the disease. Only certain patients with colorectal liver metastases can be treated by surgical resection. Different interventional treatments such as laser-induced thermotherapy, microwave ablation, as well as transarterial chemoembolization can be applied for treating colorectal liver metastases. These therapies have been discussed in various studies. However, the current medical literature is still lacking research from large long-term studies. This retrospective monocentric study includes 2140 patients with colorectal liver metastases treated by different locoregional treatments. It is based upon data collected over a period of more than 26 years at the University Hospital Frankfurt of Goethe University.

**Abstract:**

The aim of this study was to analyze the long-term results of different locoregional treatments for colorectal cancer liver metastases (CRLM), including transarterial chemoembolization (TACE), laser-induced thermotherapy (LITT) and microwave ablation (MWA). A total of 2140 patients with CRLM treated at our department between 1993 and 2020 were included in this retrospective study. The patients were divided into the following groups: LITT (573 patients; median age: 62 years), TACE + LITT (346 patients; median age: 62 years), MWA (67 patients; median age: 59 years), TACE + MWA (152 patients; median age: 65 years), and TACE (1002 patients; median age: 62 years). Median survival was 1.9 years in the LITT group and 1.7 years in the TACE + LITT group. The median survival times in the MWA group and TACE + MWA group were 3.1 years and 2.1 years, respectively. The median survival in the TACE group was 0.8 years. The 1-, 3-, and 5-year survival rates were 77%, 27%, and 9% in the LITT group and 74%, 18%, and 5% in the TACE + LITT group, respectively. The corresponding survival rates were 80%, 55%, and 33% in the MWA group, 74%, 36%, and 20% in the TACE + MWA group and 37%, 3%, and 0% in the TACE group, respectively. The long-term results of this study demonstrate the efficacy of locoregional treatments in treating patients with CRLM. The longest survival was found in the MWA group, followed by the combination therapy of TACE and MWA.

## 1. Introduction

Colorectal cancer (CRC) is one of the most commonly diagnosed cancers worldwide with a total of more than 1.9 million new diagnosed cases and 935,000 cases of death in 2020 [1]. CRC metastasizes mostly to the liver [2,3] followed by the lungs [3]. During the course of the disease, around 25% of patients with CRC develop liver metastases [4]. For patients with resectable colorectal liver metastases (CRLM), surgery and systemic chemotherapy are currently considered as the gold standard [5]. However, only a minority of patients are eligible candidates for resection [6].

In cases of unresectable liver metastases, image-guided minimally invasive interventional procedures can be applied to treat tumors locally [7]. Interventional treatments for liver metastases include thermal ablation such as radiofrequency ablation (RFA) [8,9], microwave ablation (MWA) [8,9] and laser-induced thermotherapy (LITT) [10]. Transarterial chemoembolization (TACE) is also an interventional treatment which can be performed to treat patients with CRLM [11].

While LITT and MWA are both hyperthermal treatments [12,13], which gained popularity in the management of different tumors owing to their multifarious advantages [13], TACE is a catheter-based treatment, where anticancer drugs and embolic agents are injected into the tumor-feeding vessels [14,15].

The cases of patients with CRC should be thoroughly discussed in a multidisciplinary tumor board (MDT), especially in cases of metastatic disease, due to the complexity and variety of the treatments for these patients. These MDT should include gastroenterologists, oncologists, surgeons, radiologists, radiation oncologists, and pathologists [16,17]. According to the German guidelines, FOLFIRI, FOLFOX or FOLFOXIRI regimes should be applied as first-line chemotherapies in cases of good general condition [16]. However, in cases of unresectable metastases or poor general condition, these guidelines recommend thermal ablation such as RFA for CRLM and locoregional treatments including selective internal radiation therapy (SIRT) when other treatments are not eligible [16]. The guidelines of the ESMO (European Society for Medical Oncology) state that thermal ablation should be especially considered for small-sized CRLM as well as for recurrent CRLM after initial surgical resection. Locoregional treatments such as TACE, hepatic arterial infusion chemotherapy (HAIC) and SIRT should be used for patients with CRLM in non-curative intention [17].

We recently published our long-term results on interventional treatments of hepatocellular carcinoma [18] and liver metastases from breast cancer [19]. The goal of this retrospective study was to analyze the long-term results of locoregional therapies for CRLM with a focus on LITT, MWA, and TACE in different treatment settings.

## 2. Materials and Methods

### 2.1. Ethical Statement

This retrospective monocentric study was approved by our institutional review board (approval code: 2021–202 and date of approval: 14 May 2021).

### 2.2. Patient Cohort

We enrolled a total of 2140 patients (1364 men and 776 women) with CRLM, who were treated at our department using different locoregional treatments (TACE, LITT, and MWA) between 1993 and 2020. The inclusion criteria were patients with CRC and only CRLM as distant metastases, who received interventional treatments for their hepatic metastases. We excluded patients who were treated by both ablative modalities (LITT and MWA) in order to accurately investigate the outcome of each treatment separately. The patients were divided depending on the performed treatments into five main groups as follows: (1) LITT only, (2) TACE + LITT, (3) MWA only, (4) TACE + MWA, and (5) TACE only. The patients who were treated by ablation as monotherapy (LITT only or MWA only) had CRLM with a size of ≤5 cm and a maximum number of ≤5 metastases. The patients in the combination groups of (TACE + LITT or TACE + MWA) had larger metastases > 5 cm and/or >5 metastases, which were treated by ablation after being successfully downsized by TACE.

The patients who underwent TACE as monotherapy had the most advanced stage of the disease and were not able to be downsized, thus they could not receive further treatment by ablation.

### 2.3. Statistics

Statistical analysis was performed using the program ‘Statistical Package for the Social Sciences’ (SPSS) version 22 and R package version 4.1. The main parameter of this study was survival time, which was calculated from the date of the first therapy session until death or last contact. The Kaplan–Meier method was used for the calculation of survival time. The differences between the groups were determined by the logrank test. A *p*-value of less than 5% was considered statistically significant. Cox regression analysis was used as to determine different predictors for survival time such as therapy, sex, and age.

## 3. Results

### 3.1. Treatments

A total of 573 patients (385 men and 118 women; median age: 62 years) were treated by LITT alone in 1363 treatment sessions (2.4 LITT sessions per patient).

In the combination group of TACE + LITT, a total of 839 LITT sessions (2.4 LITT sessions per patient) and 1979 TACE sessions were performed (5.7 TACE sessions per patient) in 346 patients (231 men and 115 women; median age: 62 years). The complication rate was 14.1% in the LITT group and 17.4% in the TACE + LITT group. Pleural effusion and hematoma were the most common complications in both LITT groups.

A total of 67 patients (37 men and 30 women; median age: 59 years) were treated by MWA as monotherapy in 103 sessions (1.5 MWA sessions per patient). The group of TACE + MWA included a total of 152 (93 men and 59 women; median age: 65 years), who were treated by 402 MWA sessions (2.6 MWA sessions per patient) and 1035 TACE sessions (6.8 TACE sessions per patient).

The complication rate was 2.9% in the MWA monotherapy group and 0.7% in the combination group of TACE + MWA. The most common complications were subcapsular hematomas and abscess.

A total of 1002 patients (618 men and 384 women; median age: 62 years) were treated with 4321 TACE sessions (4.3 TACE sessions per patient) as monotherapy. Table 1 shows an overview of performed treatments and complications.

### 3.2. Survival

The median and mean survival times were 1.9 years and 2.9 years in the LITT group, respectively. The 1-, 3-, and 5-year survival rates were 77%, 27% and 9%, respectively. In the combination group of TACE + LITT, the median and mean survival times were 1.7 years and 2.1 years, respectively. The 1-, 3-, and 5-year survival rates were 74%, 18% and 5%, respectively.

The median and mean survival times were 3.1 years and 7.6 years in the MWA group, respectively. The 1-, 3-, and 5-year survival rates were 80%, 55% and 33%, respectively.

The median and mean survival times were 2.1 years and 4.5 years in the TACE + MWA group, respectively. The 1-, 3-, and 5-year survival rates were 74%, 36% and 20%, respectively.

**Figure 1:**
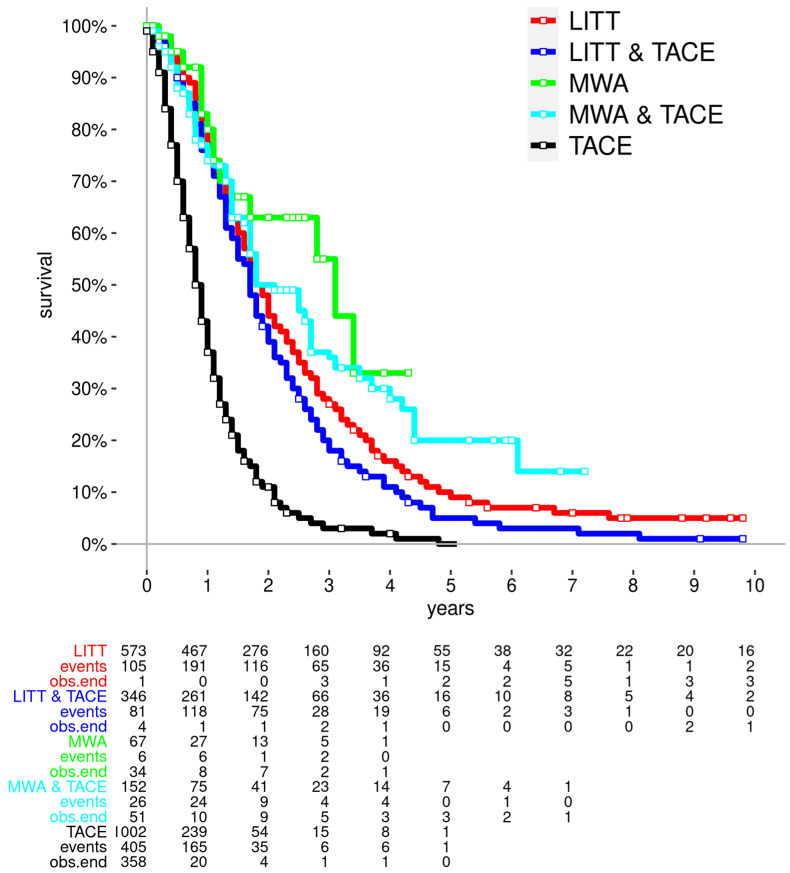
Kaplan–Meier curves for all different treatment groups and list of events along the timeline. Data for number of patients at risk, number of events (deaths), and number of patients where the observation ended (obs.end). LITT = laser-induced thermotherapy, MWA = microwave ablation, TACE = transarterial chemoembolization.

The median and mean survival times were 0.8 year and 1 year in the TACE group, respectively. The 1-, 3-, and 5-year survival rates were 37%, 3% and 0%, respectively. Figure 1 shows the Kaplan–Meier survival curves of the groups. The white dots represent the censored cases. Table 2 shows the survival of the patients in all groups. Table 3 shows a comparison of survival for different groups.

Cox regression analysis including all treatments adjusted by the age and sex of the patients shows that the chosen treatment method is the statistically significant predictor for survival, even when the age and sex of patients are added to the analysis (Table 4).

The predictive values of different treatment protocols without adjustment for age and sex of the patients as multiple univariate models are shown in Table 5, where every treatment setting is compared with the rest of the other treatments and tested regarding prediction for survival.

The Cox regression analysis after adjustment for age and sex of the patients as a multivariate statistical model is shown in Table 6. TACE as monotherapy was excluded because of algorithm restrictions. As potential predictors for survival, four treatment protocols in addition to age and female sex of patients are simultaneously included in this multi-variable model. This analysis shows that the four included treatment methods are relevant and significant predictors for survival.

## 4. Discussion

Interventional locoregional treatments including TACE, radioembolization, and thermal ablation have been used successfully in the management of CRLM [20].

To our knowledge, this is the first retrospective single-center study over a relatively long period of time. This paper aims to point out the achievements made in interdisciplinary interventional management over the past 26 years (1993–2020), considering the survival of patients with CRLM treated by different interventional locoregional treatments as well as various treatments protocols and settings.

Comparing the 3-year survival rates of MWA and MWA + TACE (55% and 36%) with LITT and LITT + TACE (27% and 18%), the improvement of MWA-based treatments is obvious, at least in this cohort. Comparisons of 5-year survival rates for MWA and TACE + MWA (33% and 20%) with LITT and LITT + TACE (9% and 5%) also support this finding. After performing various Cox regression analyses, we found that the chosen treatment protocol is a significant predictor for survival.

There are several studies that have evaluated interventional treatments including thermal ablation [21,22,23,24,25,26] and chemoembolization [27,28] for CRLM. The survival time ranged from 7.7 months to 54.4 months [21,23,24,25,26,27,28]. The 1-year survival rate ranged from 91.6% to 98.9% [22,25,26], the 3-year survival rate from 35% to 68.1% [21,22,24,25,26], and the 5-year survival rate from 17% to 48.2% [21,22,25,26].

Groeschl et al. evaluated MWA for different primary and secondary hepatic tumors including CRLM in their multicenter study [21]. The median survival time was 32.1 months. The 3- and 5-year survival rates were 45% and 17%, respectively. The survival time in our MWA monotherapy group was slightly longer.

Lee et al. compared in their study RFA with surgical resection as curative treatments for patients with CRLM [22]. The 1-, 3-, and 5-year survival rates were 92.2%, 62.4%, and 48.2% in the RFA group, respectively. These rates are higher than our survival rates in both groups of ablations as monotherapy (LITT only or MWA only). The reason for this difference in the survival may be because of the higher tumor burden in our patient cohort, since Lee et al. excluded patients with a size of >3 cm and a number of CRLM ≥ 5.

Thermoablative treatments combined with TACE showed significantly better survival in comparison to monotherapy with TACE alone. We assume that the reason for this significant difference is that the patients, who were treated only by ablation or by TACE combined with ablation, had a lower hepatic tumor burden than patients treated with TACE as monotherapy. Even if it is not always possible, this means that downsizing by TACE should be sought in order to perform ablation and achieve better outcomes.

Puls et al. included in their study a total of 87 patients with 180 CRLM, who were treated by laser ablation [23]. They excluded patients with >5 metastases or patients with metastases >5 cm. The mean survival time after the first treatment was 31.1 months. The survival time in our LITT group was 2.9 years (34.8 months).

Pacella et al. included in their prospective study a total of 44 patients with 75 unresectable CRLM, who were treated by laser ablation [24]. Regarding size, in case of single metastasis the size was up to 10 cm and in case of multiple CRLM the size was not larger than 6 cm. They did not report any major complications and found that survival in patients with complete response was significantly longer than in patients with minor or partial response. The 3-year survival rate was 35% among patients with complete response. In our study, the 3-year survival rate was 27% in the LITT group and 18% in the TACE + LITT group.

In a study by Dijkstra et al., thermal ablation was compared with partial hepatectomy for recurrent CRLM [25]. The complication rate was 19.2% in the ablation and 32% in the resection group. They reported a median survival time of 54.4 months in the ablation group and 49.2 months in the resection group. However, the difference in the complication rates and survival between both groups were not significant.

In a more recent study by Dijkstra et al. the outcomes of patients with small-size CRLM (0–3 cm) were compared with patients with intermediate-size CRLM (3–5 cm), who underwent thermal ablation (RFA and MWA) [26]. The patients with small-sized CRLM had superior survival time in comparison to the patients in the intermediate-sized CRLM. However, the difference was not significant.

Albert et al. [27] included a total of 121 patients with CRLM, who were treated by 245 chemoembolizations. They reported a median survival time of 9 months starting at the treatment. Hong et al. [28] compared TACE with yttrium-90 radioembolization as salvage treatments for patients with CRLM. A total of 21 patients were treated by TACE and 15 patients by radioembolization. The median survival time was higher in the TACE group at 7.7 months compared to the radioembolization group at 6.9 months. However, the difference between both groups was not significant. The median survival time in our TACE group was 0.8 years, which is similar to the studies of Albert et al. [27] and Hong et al. [28].

In our current study, the longest survival time was found in the MWA group, followed by the combination therapy of TACE and MWA. This highlights the importance and high relevance of these treatments.

A recent review [29], which included the studies of Wu et al. [30] and Yamakado et al. [31] among others, showed that the combination of ablation and transarterial treatments for large liver metastases (>3 cm) is effective and safe. Wu et al. [30] included a total of 30 patients with 43 CRLM (size: 1.4–10 cm), who were treated by the combination therapy of TACE and MWA. They reported a median survival time of 11 months. In the prospective multicenter study of Yamakado et al. [31] a total of 25 patients with 38 CRLM (size: 1–4.2 cm) were enrolled, who were treated by the combination therapy of TACE and RFA. The patients were not eligible for surgery and had 3 ≤ lesions with a size of ≤3, or a single lesion of ≤5 cm. They reported a median survival time of 48.4 months. The median survival time in our TACE + MWA group was 2.1 years. We believe that the reason for the differences between these studies and our study is the different tumor burdens in the patient cohorts.

## 5. Limitations

There were several limitations for this study. This study was limited by the retrospective design. Because of this, many factors were not available such as the mutation status of CRC, the exact size, number, and location of the treated CRLM, as well as tumor markers. These factors may have impact on the survival of the patients. Furthermore, progression-free survival could not be calculated. Also, this study did not include a control group in order to compare the locoregional treatments with other options such as surgery. This study showed the results over a very long period, which can be seen as an advantage. However, during this long period the interventional techniques for the treatment of CRLM may differ and change over time. A comparative prospective long-term study for the evaluation of interventional treatments is surely needed and remains important for a better analysis.

## 6. Conclusions

This study showed the efficacy and safety of several interventional modalities in different treatment protocols and settings for CRLM over a long period of time. The best survival was reported in the MWA group followed by the combination of TACE and MWA. These promising results highlight the clinical importance of both treatment modalities in the management of CRLM. We showed that patients who had a high tumor burden and were treated only by TACE had the worst outcome. However, this shows the significant role of TACE in downsizing and reducing the tumor burden, in order to allow further treatment to be performed by ablation and the achievement of better outcomes.

## Figures and Tables

**Table 1:** Treatments and complications.

Variable	LITT	TACE + LITT	MWA	TACE + MWA	TACE	TOTALS
number of patients	573	346	67	152	1002	2140
Men	385	231	37	93	618	1364
Women	188	115	30	59	384	776
Number of ablations treatments	1363	839	103	402		2707
Average number of ablation treatments per patient	2.4	2.4	1.5	2.6		
Number of TACE cycles		1979		1035	4321	7335
Average number of TACE sessions per patient		5.7		6.8	4.3	
Complication rate (%)	14.1	17.4	2.9	0.7	N/A	
Median age (years)	62	62	59	65	62	

Note: LITT = laser-induced thermotherapy, MWA = microwave ablation, TACE = transarterial chemoembolization, N/A = not available.

**Table 2:** Mean and median survival times as well as the 1-, 3-, and 5-year survival rates with 95% confidence intervals of different treatment groups.

Variable		LITT	TACE + LITT	MWA	TACE + MWA	TACE	TOTALS
number of patients		573	346	67	152	1002	2140
events		544	334	15	68	618	1579
mean survival time (years)		2.9	2.1	7.6	4.5	1.0	2.1
median survival time (years)		1.9	1.7	3.1	2.1	0.8	1.3
	median lower CI	1.7	1.6	1.7	1.7	0.8	1.3
	median upper CI	2.0	1.8		2.7	0.9	1.4
1-year survival rate		77	74	80	74	37	61
	1-year 95% lower CI	74	69	67	66	33	59
	1-year 95% upper CI	81	78	94	82	41	63
3-year survival rate		27	18	55	36	3	17
	3-year 95% lower CI	23	14	34	25	2	15
	3-year 95% upper CI	30	22	76	46	5	19
5-year survival rate		9	5	33	20	0	6
	5-year 95% lower CI	7	2	6	10	0	5
	5-year 95% upper CI	12	7	60	31	1	7

Note: LITT = laser-induced thermotherapy, MWA = microwave ablation, TACE = transarterial chemoembolization.

**Table 3:** Comparison of survival for different groups.

#	Method 1		Method 2	*p*-Value	*n* (Total)	*n*1	*n*2
a	LITT	vs.	TACE + LITT	*p* = 0.001	*n* = 919	573	346
b	LITT	vs.	MWA	*p* = 0.063	*n* = 640	573	67
c	LITT	vs.	TACE + MWA	*p* = 0.077	*n* = 725	573	152
d	LITT	vs.	TACE	*p* < 0.001	*n* = 1575	573	1002
e	TACE + LITT	vs.	MWA	*p* = 0.008	*n* = 413	346	67
f	TACE + LITT	vs.	TACE + MWA	*p* = 0.001	*n* = 498	346	152
g	TACE + LITT	vs.	TACE	*p* < 0.001	*n* = 1348	346	1002
h	MWA	vs.	TACE + MWA	*p* = 0.293	*n* = 219	67	152
i	MWA	vs.	TACE	*p* < 0.001	*n* = 1069	67	1002
j	TACE + MWA	vs.	TACE	*p* < 0.001	*n* = 1154	152	1002

Note: LITT = laser-induced thermotherapy, MWA = microwave ablation, TACE = transarterial chemoembolization. n1: number of patients of treatment method1; n2: number of patients of treatment method 2.

**Table 4:** Cox regression analysis adjusted by age of patients and sex (female) (*n* = 2140, model: *p* < 0.001, R² = 0.112).

Predictor	*p*-Value	Hazard Ratio	Lower 95%-CI	Upper 95%-CI
Method	0.000	1.289	1.250	1.330
Age	0.668	0.999	0.994	1.004
Female	0.712	0.980	0.883	1.089

**Table 5:** Cox regression analysis without adjustment by age and sex of the patients as multiple univariate models.

Predictor	*p*-Value	Hazard Ratio	Lower 95%-CI	Upper 95%-CI
LITT	0.000	0.587	0.528	0.653
TACE + LITT	0.016	0.862	0.764	0.973
MWA	0.001	0.413	0.248	0.687
TACE + MWA	0.000	0.551	0.432	0.702
TACE	0.000	3.007	2.693	3.359

Note: LITT = laser-induced thermotherapy, MWA = microwave ablation, TACE = transarterial chemoembolization.

**Table 6:** Cox regression analysis adjusted by age and sex of the patients as a multivariate model (*n* = 2140, model: *p* < 0.001, R² = 0.1606).

	*p*-Value	Hazard Ratio	Lower 95%-CI	Upper 95%-CI
LITT	0.000	0.319	0.282	0.362
TACE + LITT	0.000	0.396	0.345	0.455
MWA	0.000	0.194	0.116	0.324
TACE + MWA	0.000	0.254	0.197	0.327
Age	0.977	1.000	0.995	1.005
Female	0.954	0.997	0.898	1.107

Note. LITT = laser-induced thermotherapy, MWA = microwave ablation, TACE = transarterial chemoembolization.

## Data Availability

The data may be requested from the corresponding author. All requests must be justified and will be checked according to privacy and possible ethical restrictions.
